# Dopamine transporter phosphorylation site threonine 53 regulates substrate reuptake and amphetamine-stimulated efflux

**DOI:** 10.1186/2050-6511-13-S1-A45

**Published:** 2012-09-17

**Authors:** Jae-Won Yang, James D Foster, Amy E Moritz, Sathyavathi ChallaSivaKanaka, Margaret A Smith, Marion Holy, Kyle Wilebski, Roxanne A Vaughan, Harald H Sitte

**Affiliations:** 1 Institute of Pharmacology, Center for Physiology and Pharmacology, Medical University of Vienna, 1090 Vienna, Austria; 2 Department of Biochemistry and Molecular Biology, University of North Dakota, School of Medicine and Health Sciences, Grand Forks, ND 58202 , USA

## Background

In the central nervous system, levels of extraneuronal dopamine are controlled primarily by the action of the dopamine transporter (DAT). Multiple signaling pathways regulate transport activity, substrate efflux, and other DAT functions through currently unknown mechanisms but presumably by oligomerization, protein-protein interactions and post-translational modification, such as phosphorylation. DAT is phosphorylated by protein kinase C within a serine cluster at the distal end of the cytoplasmic N-terminus, while recent work in model cells revealed proline-directed phosphorylation of rat DAT at membrane proximal residue Thr53. However, specific phosphorylation sites in native DAT under basal condition with associated functional properties have not been ascertained so far.

## Methods

We (i) applied mass spectrometry in rodent striatal tissue and heterologous cell systems to identify *in vivo* phosphorylation sites (ii) generated a phospho-specific antibody (pT53Ab) for the confirmation of the identified phosphorylation site and for the determination of a stoichiometry of phosphorylation, involvement of PKC and phosphatase. Functional implications of this identified phosphorylation site have been tested by dopamine uptake and amphetamine-stimulated substrate efflux.

## Results

Phosphorylation of Thr53 (pThr53), occurred with a stoichiometry of ~50% under basal condition in rat striatal tissue, was unambiguously identified by mass spectrometry and immunoassay with phospho-specific antibody. pThr53 was strongly increased by phorbol esters and protein phosphatase inhibitors. Mutations of Thr53 to alanine to mimic dephosphorylation reduced dopamine transport V_max_ and ablated amphetamine-induced substrate efflux.

## Conclusions

DAT is constitutively phosphorylated at Thr53 and its phosphorylation/dephosphorylation status plays a role in the transport mechanism, particularly in dopamine uptake and amphetamine-stimulated substrate efflux.

